# My Struggle with a Tape-worm

**Published:** 1880-01

**Authors:** G. B. Pratt

**Affiliations:** Late Resident Physician of the King’s County Hospital, Brooklyn; Elkhart, Ind.


### Clinical Reports.

### NOTES FROM PRIVATE PRACTICE.

### ARTICLE VI.

*My Struggle with a Tape-worm.*

Tape-worms are, comparatively speaking, quite a common affection in most parts of the United States. Probably every practitioner of medicine has been called upon to treat more than one case of this, sometimes, troublesome disorder, and having successfully removed the worm, pins his faith to whatever drug he may have used. The removal of a tape-worm creates quite a little sensation among the friends of the party thus relieved, and the fortunate doctor finds his office besieged by a crowd of curious people and gains quite a little notoriety thereby. It is not then to be wondered at that on attempting a second time to dislodge one of these pests, he recalls to mind his former success, and tries as nearly as may be to follow the same plan of treatment

That the doctor's efforts are usually crowned with success is true, but it is also a fact that in many cases the results are very unsatisfactory, and the patient becoming weary, after repeated trials, calls another physician. This is apt to make a young practitioner feel somewhat discouraged, and it is additionally disheartening if the case happens to fall into the hands of some one with whom he is not on friendly terms, who secures the prize and gains credit through his failure. My struggle with a tape-worm will perhaps be of interest to some of my brother practitioners, and, I trust, suggest to them a readily accessible, though not a new plan of treatment.

A lady called at my office to consult me in regard to her little girl, about seven years of age, who had been complaining for several months. She explained to me that one or two doctors in the city had prescribed for the child several times, but their medicine seemed to do her no good—in fact, she was growing weaker every day. The little girl was very slender, and her face wore an anxious expresssion; her appetite, the mother informed me, was ordinarily very poor, though at times she ate ravenously. For about a week her bowels had been quite loose. "What was the character of her passages?" I asked. "Oh, I did not notice particularly," she replied, "only occasionally pieces of something white, like curdled milk; she is very fond of milk." The stools were examined, and links of tænia discovered. The parents were very much alarmed when this was made known to them, and were anxious to have that "awful thing" taken away as soon as possible. This I promised to do.

It having been some time since I had had a case of this kind to treat, and wishing to proceed against this hidden foe in as scientific a manner as possible, and according to the latest and most approved methods of warfare, I selected from my library several works which I knew treated to a greater or less extent on this subject, and settled myself to a thorough examination of them. I threw all former views to one side, and, wholly unprejudiced, searched earnestly and anxiously for the drug which was to cause this trespasser on private grounds to move on.

I first took up "Stillé's Materia Medica," and turning to the index of therapeutics, under the heading of "Tænia" I read: "acidum sulphuricum, æther, antimonii et potassii tartras, brayera, chenopodium, filix mas, granatum, kamula, oleum olivæ, oleum tiglii, pepo, stanni pulvis, tanacetum, turpentine."

I must confess to a feeling of faintness at sight of such long list of ammunition from which to select. To my mind this array included some articles as powerless as a blank cartridge, and others which, comparatively speaking, were like a charge of dynamite. However, I read what Stillé had to say in favor of each drug, and coming down to filix mas I came across this sentence, "Male fern and its preparations are almost exclusively used for the cure of tape-worm." I kept my finger on this and finished the list. Stillé's remark in regard to male fern haunted me. If this drug was used "almost exclusively" it must be the best, and if it was the best then had I found what I was in search of. But ever careful to be in the right, I opened Ringer's Therapeutics to learn his opinion, and found "Male fern is generally considered the fittest treatment for tape-worm."

Not yet satisfied, I turned to "Diseases of the Intestines and Peritoneum," Wood's Library, and here Ransom had written, "The male fern is perhaps the oldest and most widely known vermifuge, and of late has grown into much favor." Bennett in his Clinical Lectures said, "Of all the vermifuge remedies proposed for the expulsion of tape-worn, I have found the male shield fern the most effectual." Speaking of this drug Phillips (Materia Medica), wrote, "Its value in cases of tape-worm can hardly be overestimated." And Watson in his "Practice of Physic," "But the best and most trustworthy of all, in my judgment, is male shield fern." What more could I want? All these writers pronounced the same verdict. I was satisfied, but espying Flint's "Practice of Medicine" in my book-case, I took it down and found him testifying as follows: "The male fern is a tænifuge, the efficacy of which rests upon the testimony of many observers of large experience. In my own experience it has proved promptly efficacious." Already decided in favor of filix mas, this added testimony made me only the more firm, and I immediately resolved to clear the ground for action.

Having read in some of my books that, if I would make the result doubly sure, I must starve the patient for a few days, I put the little girl on the lightest known diet, viz: beef-tea. I at first thought of putting her on a water diet, but wishing to give the male fern a fair chance adopted the beef-tea. I permitted her to have nothing else to eat for forty-eight hours, and on the evening of the second day gave the famished child a full dose of castor oil. The following morning, not allowing her any breakfast, she was given a dessert-spoonful of an equal mixture of the liquid extract of male fern and mucilage. Learning that the stomach tolerated this dose, and feeling confident that now, having followed directions to the very letter, the enemy would not be long in showing the white flag (himself), I was happy, and going to make a call on an especially aggravating chronic case, I surprised this patient by my unusually jovial disposition. Returning to our little friend about four hours after the first dose was taken, and finding no apparent result, I gave a second dose and laughing away the doubts of the parents, told them to let me know when the tape-worm had passed.

In the evening the father called at the office and said that, with the exception of one important county, the returns were all in. I told him it was most too early to expect returns from back counties. He went home. As nothing resulted from this effort, I told the parents that possibly the medicine was not quite so fresh as it might be, and they should let the child resume its regular diet, and we would try again in a few days. I also prescribed a tonic mixture to be taken three times a day.

At the expiration of two weeks I repeated the procedure, with precisely the same result. This was certainly disheartening. The little girl looked as though, after due consideration, she had decided to keep her tape-worm. I was afraid to increase the dose or even try again, lest I should be under the painful necessity of making an autopsy to obtain my specimen. I had lost faith in filix mas. However, I assured the anxious parents that, being satisfied the mild means we were using were not going to have the desired result, we would have to resort to something different.

Fresh pumpkins were coming into market; procuring one of these, I had a part of the seeds deprived of their outer envelope and beaten into a paste, with fine sugar. Three weeks after the last attempt, the child was permitted to eat as much of this paste as it could be induced to take, without any previous preparation whatever. The day following I received word from the mother that the worm was being passed, and wanting to know what she should do. I replied, "Let it pass." In the afternoon the father came into the office, looking pale and haggard; he seemed older by ten years than when I saw him the day before. He grasped the back of a chair for support, and appeared to be laboring under some terrible shock to the nervous system.

"Why, my dear fellow!" I exclaimed, "what is the matter; what has happened?" He raised his eyes to mine for a moment, and dropping them, said, in a voice shaking with emotion, "It is gone." "Gone!" I cried; "what, the child?" He glanced quickly up at me, and with a faint smile playing over his countenance, answered, "No, the worm."

And so it proved; the tape-worm had changed its mind, and not being pleased with its cool reception, had drawn its long chain of white links back into the child's intestines. But another hearty meal of the pumpkin seed paste brought away the intruder, head and all. And ere long, lest he should escape us a second time, we had fifty feet of tænia solium floating peacefully in equal parts of alcohol and water.

G. B. PRATT, M.D.,
*Late Resident Physician of the King's County Hospital, Brooklyn.*

ELKHART, IND.

---

SALICYLATE OF IRON.—Dr. Wallis White (*Glasgow Medical Journal*, Aug., 1879) prepares the salicylate of iron by dissolving together sulphate of iron 1.6 grams, salicylate of soda 2.00 grams, and acetate of soda 1.3 grams, in 30 grams of water. The solution has at first a pale port-wine appearance, which darkens on exposure to the air; it has a pleasant taste, and 30 grams contain 2 grams of salicylate of iron. Its primary action seems to be to promote secretion, stimulating the skin. It does not constipate the bowels, but rather corrects the alvine secretions. As a prophylactic against septicæmia after surgical operations it is valuable. It promotes perspiration, cleans the tongue, lowers the temperature, and reduces the pulse. It may be administered with freedom and in large quantitics in cases of anæmia, without interfering with digestion. In skin diseases also, and in desquamative nephritis where the digestive organs have become weakened, and a salt of iron is indicated, its powers are very marked. Salicylate of iron seems to combine the astringent powers of the iron, but in a minor degree to the sulphate or perchloride, with the antiseptic, antipyretic powers of the salicylic acid. If the preparation is long continued, some of it passes out unchanged with the urine.—*Practitioner*, Oct., 1879.

